# Immunoneuroendocrine, Stress, Metabolic, and Behavioural Responses in High-Fat Diet-Induced Obesity

**DOI:** 10.3390/nu16142209

**Published:** 2024-07-10

**Authors:** María del Carmen Navarro, Isabel Gálvez, María Dolores Hinchado, Eduardo Otero, Silvia Torres-Piles, Javier Francisco-Morcillo, Mónica de La Fuente, Leticia Martín-Cordero, Eduardo Ortega

**Affiliations:** 1 Immunophyisiology Research Group, Instituto Universitario de Investigación Biosanitaria de Extremadura (INUBE), 06071 Badajoz, Spain; cnavarropz@unex.es (M.d.C.N.); igalvez@unex.es (I.G.); mhinsan@unex.es (M.D.H.); eoteroc@unex.es (E.O.); 2 Immunophysiology Research Group, Physiology Department, Faculty of Sciences, University of Extremadura, 06071 Badajoz, Spain; 3 Immunophysiology Research Group, Nursing Department, Faculty of Medicine and Health Sciences, University of Extremadura, 06071 Badajoz, Spain; 4 Immunophysiology Research Group, Department of Medical-Surgical Therapy, Faculty of Medicine, University of Extremadura, 06071 Badajoz, Spain; storres@unex.es; 5 Anatomy, Cell Biology and Zoology Department, Faculty of Sciences, University of Extremadura, 06071 Badajoz, Spain; morcillo@unex.es; 6 Unit of Animal Physiology, Department of Genetics, Physiology, and Microbiology, Faculty of Biology, Complutense University of Madrid, 28040 Madrid, Spain; mondelaf@ucm.es; 7 Immunophysiology Research Group, Nursing Department, University Center of Plasencia, University of Extremadura, 10600 Plasencia, Spain

**Keywords:** obesity, macrophages, inflammation, stress, anxiety, prevention, management

## Abstract

Obesity has reached global epidemic proportions, and even though its effects are well-documented, studying the interactions among all influencing factors is crucial for a better understanding of its physiopathology. In a high-fat-diet-induced obesity animal model using C57BL/6J mice, behavioural responses were assessed through a battery of tests, while stress biomarkers and systemic inflammatory cytokines were measured using an Enzyme-Linked ImmunoSorbent Assay and a Bio-Plex Multiplex System. The peritoneal macrophage microbicide capacity was analysed via flow cytometry, and crown-like structures (CLSs) in white adipose tissue (WAT) were evaluated through staining techniques. Results indicated that obese mice exhibited increased body weight, hyperglycaemia, and hyperlipidaemia after 18 weeks on a high-fat diet, as well as worse physical conditions, poorer coordination and balance, and anxiety-like behaviour. Differences in corticosterone and noradrenaline concentrations were also found in obese animals, revealing a stress response and noradrenergic dysregulation, along with a weakened innate immune response characterized by a lower microbicide capacity, and the presence of an underlying inflammation evidenced by more CLSs in WAT. Altogether, these findings indicate that obesity deteriorates the entire stress, inflammatory, metabolic, sensorimotor and anxiety-like behavioural axis. This demonstrates that jointly evaluating all these aspects allows for a deeper and better exploration of this disease and its associated comorbidities, emphasizing the need for individualized and context-specific strategies for its management.

## 1. Introduction

Obesity, a prevalent health concern globally, is associated with various physiological and psychological complications. High-fat diets have been extensively studied for their role in inducing obesity and related comorbidities such as metabolic syndrome and insulin resistance in animal models, and they have been recognized to be adequate for mirroring human obesity [1]. It has also been found in studies that diets enriched in fat and cholesterol could be related to an increase in aggression, anxiety, depression, and a decrease in exploration associated with new environments [2,3,4,5].

A long-term diet of high-fat foods can cause disorders in the lipid metabolism in the body, contributing to depressive and anxious behaviour [6,7]. This could be because lipids act as signalling molecules for structural development, the conduction of nerve impulses, neurogenesis, synapses, myelin formation, etc., performing a wide variety of physiological activities within the brain [8,9,10]. It is also thought that the neurobiological factors causing this behaviour during the consumption of a high-fat diet are associated with inflammatory factors in the central and peripheral nervous system [11,12]. Thus, some studies have shown that inflammation hinders motivated responses to something novel in humans [13] and reduces the exploration of new environments in rodents [14]. Exploratory capacity is an important aspect in the context of evolution, since it allows the animal to know the potential risks around it (predators or other dangers) and the benefits (mates, food, nesting spaces) in a new place [15].

Understanding how obesity affects motor capabilities in animal models, such as mice, is crucial for unravelling the complex underlying mechanisms and clinical implications of this disease. Behaviour is manifested through action, and such action requires motor skills, including body coordination. Obesity is associated with a low motor coordination capacity, especially in balance ability, according to studies conducted in children with obesity [16,17], indicating that a high relative weight contributes to poor motor skills, which worsen with age and weight gain [17]. Likewise, obesity has detrimental effects on muscle function, altering the locomotor capacity and metabolic cost of locomotion of individuals [18]. However, research on motor responses in obesity models has been limited to date, with a scarcity of studies addressing this specific area.

Furthermore, high-fat diets not only lead to physiological responses of weight gain and cognitive impairment [19] but can also modulate several stress and metabolic responses, as well as alterations in the immune system. Thus, it is known that the dysregulation of innate and adaptive immunity leading to chronic, low-level, tissue-specific, and systemic inflammation is one major factor that contributes towards the progression of many obesity-related disorders and metabolic diseases, such as insulin resistance leading to type 2 diabetes mellitus [20,21,22]. In this context, the immune response, and especially innate/immune cells play a pivotal role in this condition [23]. Macrophages are major components of the immune response and have been identified as key contributors to inflammation and metabolic dysregulation in white adipose tissue (WAT) in inflammatory states [20,24]. This local proliferation of macrophages occurs mainly at crown-like structures (CLSs) that surround necrotic adipocytes, resulting in a preferential increase in macrophages in WAT [25]. The consequence and impact of local AT macrophage proliferation, in the pathophysiology of AT dysfunction and metainflammation remains uncharacterized and is fertile ground for future research in the field [26].

Understanding the intricate interplay between high-fat diet-induced obesity and associated metabolic, inflammatory, and behavioural responses is crucial for the study of preventive and therapeutic strategies. However, these responses associated with obesity have been only evaluated partially or isolated in different experimental approaches and in different individuals. The objective of this study is to comprehensively evaluate the immunophysiological (interactions between the nervous and immune systems), inflammatory response, sensorimotor and anxiety-like behaviour of a murine model of high-fat diet-induced obesity. To the best of our knowledge, this is the first time that immunoneuroendocrine biomarkers, local and systemic inflammatory responses, and anxiety-like and sensorimotor behaviour are evaluated altogether in the same groups of animals with the same metabolic status. With this approach, the impact of high-fat diet-induced obesity is further explored in the complex relationship between behavioural, cognitive, motor, inflammatory, stress, and metabolic interactions, which can validate an animal model of obesity for a better understanding of this syndrome and establish new integrative therapeutic strategies. Thus, our objective is focused on validating the hypothesis regarding obesity as a syndrome in which a metabolic and immunoneuroendocrine interaction is crucially interdependent.

## 2. Materials and Methods

### 2.1. Experimental Design

Thirty-two C57BL/6J mice, including both males and females, were bred at the Animal Facility of the University of Extremadura. Upon reaching 8 weeks of age, the mice were randomly allocated to one of two dietary groups for a period of 18 weeks until sacrifice. One cohort (n = 16) was placed on a high-fat diet (HFD) (260HF diet; SAFE, Augy, France), designated as the obese group, while the other cohort (n = 16) served as the healthy control group (lean group) and received standard laboratory rodent chow (SD) (A04 diet; SAFE, Augy, France). All mice had unrestricted access to water and were individually housed in a climate-controlled environment (22 ± 1 °C; 60 ± 5% humidity) with a 12 h light/12 h dark cycle (light from 23:00 to 11:00 h; dark from 11:00 to 23:00 h). Throughout the study, mice were able to maintain olfactory and visual contact with each other to mitigate potential negative effects of isolation. Behavioural testing was conducted on both groups of animals on the final day of the diet protocol. Following a 12 h fasting period, blood samples and visceral WAT were collected from anesthetized animals for comparison between obese and lean groups.

This investigation was approved by the Bioethics Committee for Animal Experimentation of the University of Extremadura (registry number 70/2018 for project IB18011), in accordance with the National and European legislation for the protection of animals used for research.

### 2.2. Diet Protocol

At eight weeks of age, animals started receiving each specific diet. Mice received either a high-fat diet (HFD) “260HF” (SAFE, Augy, France), containing 36% fat (providing 58.8% of the total energy intake), recognized for being suitable for the study of obesity and its associated complications in mice [20]; or a standard diet “A04” (SAFE, Augy, France), which contains approximately 5% lipids, with 8% of the energy derived from fat. All animals remained on their respective diet throughout the study, until sacrifice 18 weeks later.

To mitigate potential gastric damage resulting from an abrupt dietary transition, a combination of both diets (50% standard feed and 50% high-fat feed) was provided during the first week of intake. Lean animals were provided with 40 g of standard feed weekly, and obese animals had 30 g of high-fat feed per week. Weekly food intake and body weight were closely monitored for all mice to ensure adherence to the dietary protocol and to track changes in weight over time.

### 2.3. Physical Condition, Sensorimotor and Behavioural Tests

Tests were performed the day before sacrifice day, during the activity period of rodents (dark period) at approximately 26 weeks of age. All tests were conducted with the room’s lights off, and the sole source of illumination was a red-light bulb.

#### 2.3.1. Tests Assessing Physical Condition

##### Grip Strength Test

Grip strength was measured with the Grip Strength Test (BIO-GS3, Bioseb, Chaville, France) following the procedure outlined by Cabe and colleagues [27]. To measure grip strength in the front paws of the mice, the experimenter held the mouse gently by the base of the tail, allowing the animal to grasp the apparatus with its front paws. Then, the animal was placed in the horizontal plane, parallel to the sensor, pulling it backward by the tail. The force applied just before losing grip was recorded as the maximum tension. The test was performed in triplicate, and the result was calculated as the mean of the three measurements divided by the weight of the animal. The final result was referred to as the force value.

##### Tight-Rope Test

The apparatus consisted of a 60 cm long hemp rope, divided into segments of 10 cm and elevated to a height of 40 cm, positioned horizontally over a bed of shavings. The test began by placing the animal in the centre of the tight-rope. The maximal testing time was 30 s and was performed only once per individual.

The first aim of this test was to assess neuromuscular vigour. For this purpose, we focused on the parameter “percentage of falls”. Additionally, this test was also used to evaluate the coordination of the animals as a sensorimotor response, for which the parameter “percentage of animals that complete the test” was analysed.

#### 2.3.2. Tests Assessing Sensorimotor Response

##### Wood Rod Test

Balance and motor coordination were assessed with this test. Following the protocol outlined by Baeza and colleagues [28], the test entailed a wood rod measuring 2.9 cm in width and 80 cm in length, suspended in the air with the assistance of two bases, one on each side, at a height of 22 cm, positioned above of a bed of shavings and divided into segments of 10 cm. The test began by placing the animal in the centre of the wood rod and concluded after 20 s or if the animal managed to reach one of the ends. The test was conducted in duplicate. The parameter selected to evaluate the balance was the “percentage of mice that complete the test” and to assess the motor coordination, the “percentage of mice to cover at least 1 segment”.

##### Wire Rod Test

Similar to the wood rod test, this test consisted of a wire rod with a diameter of 1 cm and a length of 50 cm, positioned at a height of 30 cm, supported by two platforms at the ends. The test began by placing the animal in the centre of the wire rod and concluded after 20 s or if it managed to reach one of the ends. The test was also conducted in duplicate. The parameter “percentage of mice that complete the test” evaluated the balance and “number of segments” was used to evaluate the coordination of the animals.

#### 2.3.3. Tests Assessing Pain and Anxiety/Stress

##### Hot Plate Test

The hot plate test is commonly used to assess sensitivity to thermal pain. It is a simple and rapid test to detect the analgesic properties of different treatments in rodents or changes in pain sensitivity in response to experimental manipulations. It was evaluated using the Hot Plate LE7406 (Panlab, Barcelona, Spain). The method assessed two nociceptive parameters: pain threshold and pain latency time.

For the procedure, the mouse was placed into an open cylindrical space positioned above an activated hot plate. The initial measurement involved assessing the pain threshold. To accomplish this, the mouse was positioned on the hot plate starting from the initial temperature of 25 °C, while the temperature gradually increased with an accuracy of 0.1 °C. The temperature at which the animal exhibited a pain response, characterized by hind limb jumping or licking, was recorded as its pain threshold. Subsequently, the mouse was removed from the plate, and the second measurement was conducted. Here, the temperature of the plate was allowed to rise to 55 °C, and once this temperature was reached, the mouse was placed on the plate, and the time (in seconds) that passed until the animal exhibited one of the two aforementioned pain response behaviours was recorded as the pain latency time.

##### Elevated Plus Maze Test

The elevated plus maze test assesses the state of anxiety exhibited by the rodent, based on its aversion to height and open spaces. With two types of arms (one with high opaque walls, representing an enclosed space, and the other without walls, allowing the animal to perceive the environment and height), the time and frequency the mouse enters and spends on each arm determine the anxiety-like behaviour of the animal [29,30].

This test consisted of two open arms (without walls) measuring 50 cm in length and 10 cm in width, facing each other, and two closed arms (with walls 40 cm in height) of the same dimensions as the open arms, positioned perpendicular to the latter. It was constructed of rigid black plastic material, and was elevated 62 cm above the ground on a metal support with four legs.

The test was conducted under red light (20 W) and began by placing the animal in the central area of the apparatus, facing a closed arm. The duration of the test was 5 min. The parameter selected to evaluate the anxiety-like behaviour of the animals was the percentage of time in open arms with respect to the total time, calculated by dividing the time spent in open arms (in seconds) by the total duration of the test (300 s) and multiplying by 100, and the corresponding results were compared between lean and obese animals.

##### Hole-Board Test

Anxiety-like behaviour was also evaluated through the hole-board test. The protocol used for the hole board test was that as followed by Viveros and colleagues [31]. The hole-board apparatus comprised a square, uncovered device measuring 45 cm in height with a square base of 60 cm per side. The walls and base were of a matte black colour, divided into 36 squares, each measuring 10 cm per side and demarcated by white lines. In the central area, there were four equally spaced holes, each with a diameter of 3.8 cm. Within these holes, a circular red surface was placed. The test began by placing the animal in one of the corners. The duration of the test was 5 min, monitored via video recording and was conducted under red light.

The parameter for evaluating the anxiety-like behaviour in the hole board test was the total locomotion, measured as the number of squares crossed, and mean locomotion, calculated as the number of squares crossed and divided by the number of total squares, that is 36. The mean values obtained for each of the parameters were compared between both groups of lean and obese animals.

Furthermore, the behaviour of head-dipping or hole exploration was also evaluated. According to File and Wardill [32], the time and number of head-dippings are inversely proportional to the animals’ level of anxiety.

##### Open Field Test

This test is also commonly used to assess anxiety-like behaviour. As rodents display thigmotaxis behaviour (a tendency to move along walls), any form of locomotion characterized as “internal” or “external” (away from the walls of the testing arena, in the central and open zone) is inversely correlated with the rodents’ level of emotionality [33]. When a mouse feels threatened or stressed, it seeks protection and hiding. The way the device used for behavioural assessment is structured only allows the animal to feel safe by being in contact with the walls. Therefore, the mouse’s level of stress/anxiety is indicated by its interaction with these areas, and conversely, if its stress/anxiety level is low.

The test comprises a walled board (measuring 80 cm wide, 80 cm long, and 30 cm high) divided into 16 quadrants of 10 × 10 cm. The 12 quadrants composing the perimeter of the board are considered “external squares”, while the remaining 4 squares are labelled “internal squares”. The test duration was 5 min, monitored via video recording, and commenced by placing the animal in the centre of the board.

In this test, horizontal locomotor activity is assessed, reflecting the animal’s ambulation throughout the duration of the test and indicating the level of stress/anxiety based on its movement patterns [34]. Therefore, peripheral, central, and total ambulation were assessed (represented by the number of squares crossed on each of the areas). The mean values obtained for each of the parameters were compared between both groups of lean and obese animals.

### 2.4. Body Measurements and the Collection of Biological Samples

Body weight measurement started the first week of the protocol and continued weekly for the entire lifespan of each mouse. Fasted animals were gas anaesthetized with isoflurane, using a standard procedure (a starting dose of 3–5% isoflurane, a maintenance dose of 1.5–3% isoflurane). Biological samples were obtained from live, anaesthetized animals. Whole blood was drawn via cardiac puncture. The fasting blood glucose concentration and lipid profile including total cholesterol, high-density lipoprotein cholesterol (HDL-C), calculated low-density lipoprotein cholesterol (cLDL-C), and triglycerides (TG) were measured in whole blood (LUX^®^, Microcaya, Bilbao, Spain). Peritoneal cells were obtained by injecting 4 mL of a phosphate-buffered saline (PBS) medium into the peritoneal cavity of the live anesthetized animal, followed by the aspiration of the fluid from the cellular suspension, which was then deposited into polypropylene tubes. Visceral WAT was carefully dissected via laparotomy and then embedded in an optimal cutting temperature (OCT) compound and snap-frozen in liquid nitrogen. Until further analysis, samples were stored at −80 °C.

### 2.5. Determination of Plasma Concentrations of Norepinephrine (NA), Corticosterone (CTC), and Extracellular Heat-Shock Protein eHsp72

The determination of plasma concentrations of noradrenaline, corticosterone, and eHSP72 in the experimental groups was conducted via Enzyme-Linked ImmunoSorbent Assay (ELISA) using ELISA kits (Cusabio, Houston, TX, USA and MyBioSource, San Diego, CA, USA). For the assay, standards (used to generate the standard curve) and plasma samples were incubated in a 48-well plate, which was coated with a specific antibody for the protein of interest that binds to the protein present in the sample, eliminating unbound material through washes. A horseradish peroxidase (HRP) conjugate with the specific antibody was also added to each well. After incubation and the removal of excess conjugate by washing, a chromogenic substrate solution (TMB) was added to the wells, which reacted catalytically with the HRP conjugate, forming a coloured product. Finally, a “stop” solution was added to halt the substrate reaction, and the absorbance was measured at a wavelength of 450 nm using an ELISA plate reader (Tecan, Männedorf, Switzerland). The absorbance signal may be directly or inversely proportional to the amount of protein present in the sample, depending on the kit used. Protein concentration was determined by interpolation on the standard curve generated using values from standard samples of known concentrations, performed manually, and using MyAssays software (https://www.myassays.com/, accessed on 12 December 2022).

### 2.6. Determination of Concentrations of Systemic Inflammatory Cytokines

Plasma concentrations of inflammatory cytokines (IL-1β, IL-10, IL-6, and TNF-α) were measured using the Bio-Plex^®^ 200 system (BioRad, Hercules, CA, USA) employing the high-sensitivity kit ProcartaPlex Immunoassay kit (Invitrogen, Waltham, MA, USA). This immunoassay was based on the use of magnetic microspheres to detect and quantify various target analytes simultaneously. Magnetic microspheres were incubated with mouse plasma as the sample and anti-IL-1β, anti-IL-10, anti-IL-6, and anti-TNF-α antibodies, and labelled with Streptavidin-Phycoerythrin for subsequent visualization. To minimize inter-assay variations, all samples were analysed using the same kit on the same day.

### 2.7. Oxidative Burst Assay

The flow cytometry analysis was used to evaluate the microbicide capacity of opsonized bacteria within peritoneal macrophages. This quantitative technique enables a very accurate determination of the ability of macrophages to ingest bacteria and produce the superoxide anion (O_2_^−^, indicative of an oxygen-dependent microbicide capacity).

First, *Escherichia coli* (*E. coli*) was obtained at an optical density of 1.6 (O.D.), fixed in PFA (1%) (PanReac AppliChem, Barcelona, Spain), washed and filtered (0.22 μm diameter filter) with PBS. Subsequently, the bacteria were stained with FITC (fluorescein isothiocyanate) at a final concentration of 1 μg/mL for 30 min at 37 °C in darkness and agitation, and then washed twice with PBS. Once stained, the bacteria were opsonized by incubating them with serum for 2 h at 37 °C in darkness and agitation (800 μL of bacteria in PBS with 200 μL of serum).

Next, 200 μL of peritoneal exudate from each mouse were incubated for 1 h at 37 °C in darkness and agitation with 50 μL of opsonized *E. coli*-FITC bacteria, Hoechst 33342 (10 μg/mL), 7-Aminoactinomycin D (7AAD 1 μg/mL), 250 μL of PBS, and 2% fetal bovine serum (FBS). A control was performed using 100 μL of peritoneal exudate along with Hoechst 33342 (10 μg/mL), 7AAD (1 μg/mL), 400 μL PBS, and 2% FBS. Finally, the sample was analysed using flow cytometry (MACSQuant WYB, Miltenyi Biotec GmbH, Bergisch Gladbach, Germany) with 3 lasers (405 nm, 488 nm, 561 nm), and the results were processed and analysed using the “FlowJo data analysis” v9 software.

Results were expressed as the percentage of macrophages producing O_2_^−^ upon the ingestion of bacteria and compared between lean and obese groups.

### 2.8. Determination of Adipocyte Size and Crown-like Structures in WAT

WAT was cut into 12–15 µm sections in a cryostat (LEICA, CM 1950, Leica Biosystems, Richmond, IL, United States) at −30 °C and mounted on Superfrost^®^ Plus microscope slides (Thermo Fisher Scientific, Braunschweig, Germany). Samples were stored at −20 °C until further analysis.

For the evaluation of adipocyte size in adipose tissue, hematoxylin-eosin staining was performed on previously processed and fixed adipose tissue sections for subsequent visualization using a conventional optical microscope (Axioskop model, Zeiss, Oberkochen, Germany). The AxionVision LE visualization 4.8. software (Carl Zeiss™, White Plains, NY, USA) was utilized for observing adipocytes at 40× magnification and measuring them. To determine size, the diameter of all observable adipocytes per field was calculated across 10 randomly selected fields per mouse. The conversion of diameters from pixels to micrometres was conducted.

To evaluate CLS presence in WAT, immunohistochemistry was performed. Slides containing the adipose tissue samples were washed with PBS + Triton X-100 (Merck KGAa, Darmstadt, Germany) and then fixed with 4% paraformaldehyde (PanReac AppliChem, Barcelona, Spain) for 5 min. After a series of washes with PBS + Triton X-100 and with PBS + gelatin (PanReac AppliChem, Barcelona, Spain) + Triton X-100, non-specific binding sites were blocked with PBS + gelatin + Triton X-100 + lysine (Merck KGAa, Darmstadt, Germany) for 1 h. Finally, the antibody F4/80 (Alexa Fluor^®^ 488 Anti-mouse F4/80 Antibody, BioLegend, San Diego, CA, USA) was added at a concentration determined after titration. The samples were then incubated overnight in a humid chamber in darkness. The next day, slides were washed again with PBS + Triton X-100 and with PBS + gelatin + Triton X-100. DAPI 2 μM (Invitrogen, Darmstadt, Germany) was added as a nuclear stain. After 15 min of incubation, the slides were washed with PBS. Coverslips and Mowiol^®^ 40–88 (Sigma-Aldrich, Taufkirchen, Germany) were used for mounting the samples. The samples were stored at 4 °C in a humid chamber in darkness until they were visualized under the fluorescence microscope.

Immunostained adipose tissue sections were observed under a conventional transmitted light and fluorescence microscope (Nikon ECLIPSE 80i, Nikon, Tokyo, Japan), obtaining digital images with a camera attached to the microscope (Nikon Digital Camera DXM 1200F, Nikon, Tokyo, Japan). The images of the antibodies at different wavelengths were overlaid into a single image and optimized using Adobe PhotoShop v.CS4 software (Adobe, San Jose, CA, USA). For CLS counting, 10 randomly chosen fields of view were selected using a 40× objective.

### 2.9. Statistical Analysis

Values were expressed as mean ± standard error of the mean (SEM) followed by the 95% confidence interval (CI), where applied. The variables were normally distributed (tested using the Kolmogorov–Smirnov normality test). Student’s *t*-test was used for comparisons between the pairs of groups (paired or non-paired samples). Statistical significance was considered when the probability value “*p*” (*p*-value) was equal or less than 0.05, with three levels of statistical significance: *p* < 0.05, *p* < 0.01, *p* < 0.001, each indicating increasing levels of significance. Effect size (ES) was calculated using Cohen’s d and interpreted as very low (d < 0.2), low (0.2 < d < 0.5), medium (0.5 < d < 0.8), and high (d > 0.8).

## 3. Results

### 3.1. Analysis of Weight, Glycaemic and Lipid Profile in Obesity

**Figure 1:**
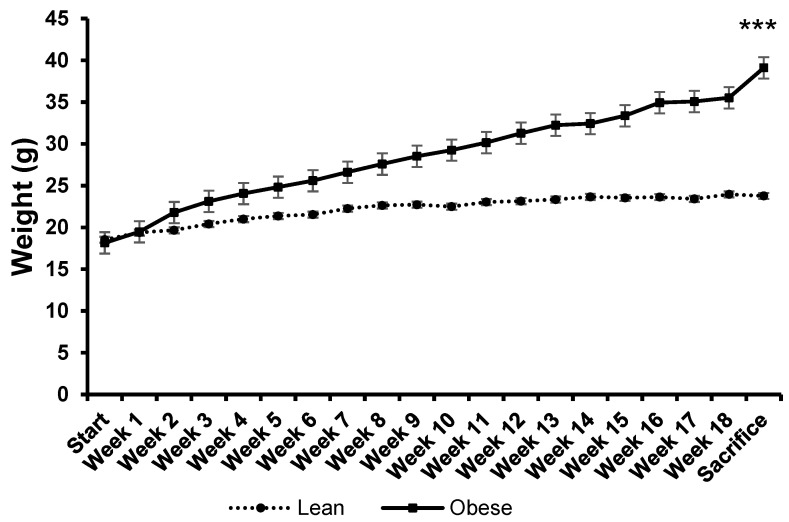
Weight progression during the standard or high-fat diet protocols. Each value represents the mean ± SEM of weights recorded weekly in 8 animals from each group during the 18 weeks of the diet protocols. Starting weight (6–8 weeks of age) and sacrifice weight (22–24 weeks of age) are indicated. Values are expressed in grams. *** *p* < 0.001 compared to the lean group on sacrifice day. ES = 1.5 (95% CI for lean: (21.4, 23), 95% CI for obese: (26, 31.2)).

**Figure 2:**
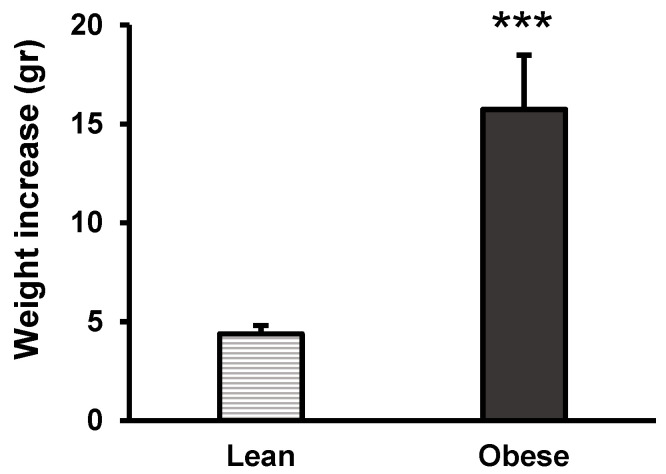
Weight increase of lean and obese mice upon the completion of the diet protocol. Values represent the mean ± SEM of the weight increases of 8 animals in each group, calculated by subtracting the initial weight of each animal from the weight on the day of sacrifice (last recorded weight). Values are expressed in grams. *** *p* < 0.001 compared to the lean group. ES = 2 (95% CI for lean: (3, 5.3), 95% CI for obese: (9.5, 22.2)).

The weight gain over 18 weeks in both groups is represented in Figure 1. In our model, the group of obese animals experienced a greater increase in weight throughout the experimental protocol compared to the lean animals. Figure 2 shows the weight increase after 18 weeks of diet in each group, revealing a three-fold increase in weight in the obese group compared to the lean group. This weight increase is due solely to the dietary intervention since the rest of the conditions were the same for both groups.

The body and metabolic parameters regarding lean and obese mice are set out in Table 1. As expected, we confirm the effectiveness of our model of HFD-induced obesity, as obese animals exhibited higher body weights and hypertrophy in their adipocytes, increased fasting glucose concentrations, and elevated levels of TG, total cholesterol, HDL-C, and cLDL-C.

### 3.2. Behavioural Tests

#### 3.2.1. Physical Condition

**Figure 3:**
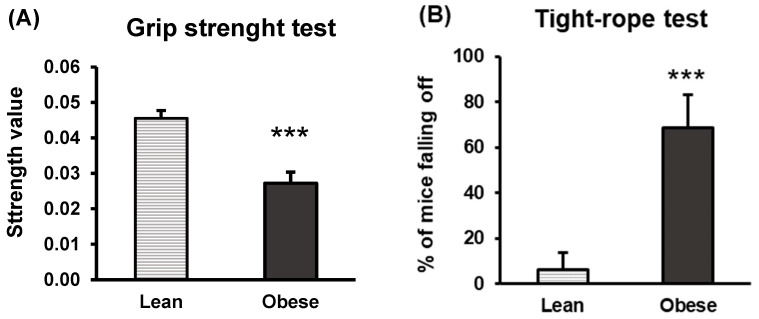
Strength and muscle vigour in lean and obese mice. Grip strength test (**A**). Tight-rope test (**B**). Each column represents the mean ± SEM of the values determined in 16 animals in each group. The strength value is calculated by dividing the average of 3 grips by the weight of each animal. *** *p* < 0.001, compared to the lean group. ES for grip strength test = 2 (95% CI for lean: (0.04, 0.05), 95% CI for obese: (0.02, 0.03)); ES for tight-rope test = 1.6 (95% CI for lean: (−7.1, 19.6), 95% CI for obese: (43.2, 94.3)).

Animals’ physical conditions were analysed using the grip strength and the tight-rope test. The values of grip strength were lower (*p* < 0.001) (Figure 3A), and the percentage of falls in the tight-rope test was higher (*p* < 0.001) (Figure 3B) in the obese group with respect to the lean group. Those results revealed that the obese animals exhibited less strength than the lean ones in both tests, showing a negative impact on the animals’ physical condition, partly due to changes in body composition that occur during the development of the obese state.

#### 3.2.2. Sensorimotor Responses

**Figure 4:**
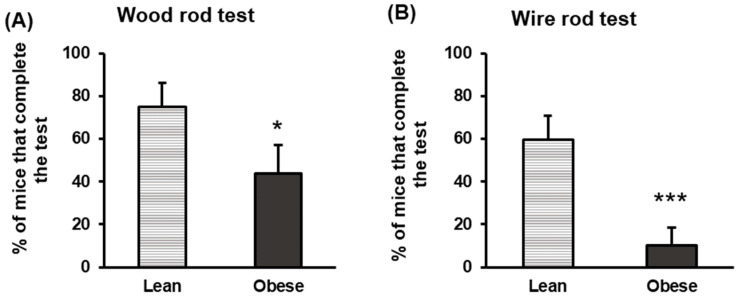
Balance in lean and obese mice. Wood rod test (**A**). Wire rod test (**B**). The percentage of animals reaching the end of the wood rod or the wire rod test within the duration of the test (20 s) is measured. Each column represents the mean ± SEM of the values obtained in 16 animals in each group. Results are expressed in percentages. * *p* < 0.05, *** *p* < 0.001, compared to the lean group. ES for wood rod test = 0.77 (95% CI for lean: (55.5, 94.5), 95% CI for obese: (20.2, 67.3)); ES for wire rod test = 1.5 (95% CI for lean: (39.4, 79.4), 95% CI for obese: (−5.5, 25.5)).

The tests that assessed balance parameters are depicted in Figure 4. The results showed that the percentage of animals that completed the test was significantly lower in the obese group compared to the lean animal group, both for the wood rod (Figure 4A) and the wire rod test (Figure 4B) (*p* < 0.05, *p* < 0.001, respectively). Thus, these results confirm that obese animals have an impaired balance with respect to lean animals.

**Figure 5:**
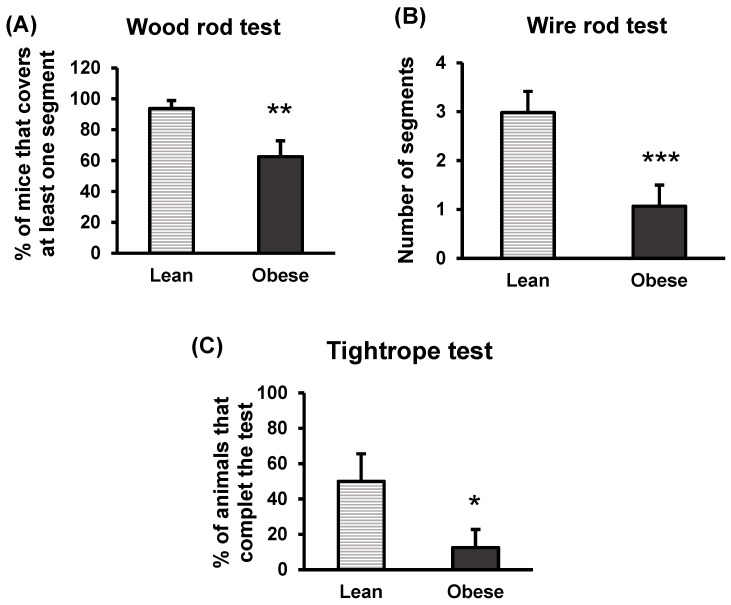
Motor coordination in lean and obese mice. Wood rod test (**A**). Wire rod test (**B**). Tightrope test (**C**). The percentage of animals performing at least one segment in the wood rod test, the number of segments traversed by the animal in the wire rod test, and the percentage of animals completing the tightrope test within the duration of each test (20 s) are measured. Each column represents the mean ± SEM of the values determined in 16 animals of each group. Results are represented in percentage, number, and percentage, respectively. * *p* < 0.05, ** *p* < 0.01, *** *p* < 0.001, compared to the lean group. ES for wood rod test = 1.2 (95% CI for lean: (84.6, 102.9), 95% CI for obese: (44.3, 80.7)); ES for wire rod test = 1.3 (95% CI for lean: (2.2, 3.7), 95% CI for obese: (0.3, 1.9)); ES tight-rope test = 0.86 (95% CI for lean: (22.5, 77.5), 95% CI for obese: (−5.7, 30.7)).

Regarding motor coordination, results revealed that a lower percentage of obese animals covered at least one segment of the rod in comparison with the lean group in the wood rod test (*p* < 0.01) (Figure 5A). In the wire rod test (Figure 5B), the obese group completed a lower number of segments (*p* < 0.001), compared to the lean animals. Finally, motor coordination was also assessed using the tight-rope test, demonstrating that a lower percentage of obese animals completed this test compared to the lean group (*p* < 0.05) (Figure 5C). In light of the results, it is highlighted that the group of obese animals exhibited impaired sensorimotor responses, showing compromised balance and motor coordination, compared to the lean animal group.

#### 3.2.3. Pain and Anxiety-like Behaviour

Obesity appears to be associated with reduced neural efficiency and slower information processing and integration [35], as well as a state of stress/anxiety [22]. In this section, we describe the results regarding the behavioural tests measuring pain and anxiety-stress levels in these animals through several tests.

##### Pain

Table 2 shows the nociceptive parameters evaluated by the hot plate test (pain threshold, or the minimum intensity at which a stimulus is considered painful, and pain latency, or the maximum time it takes for an individual to perceive the stimulus as painful). No significant differences between both groups were found. However, there was a trend towards a decrease in latency time in the obese animal group (*p* = 0.07), suggesting an increase in pain sensitivity in these mice, as they exhibited a slightly lower reaction time to this stimulus.

##### Elevated Plus Maze Test

**Figure 6:**
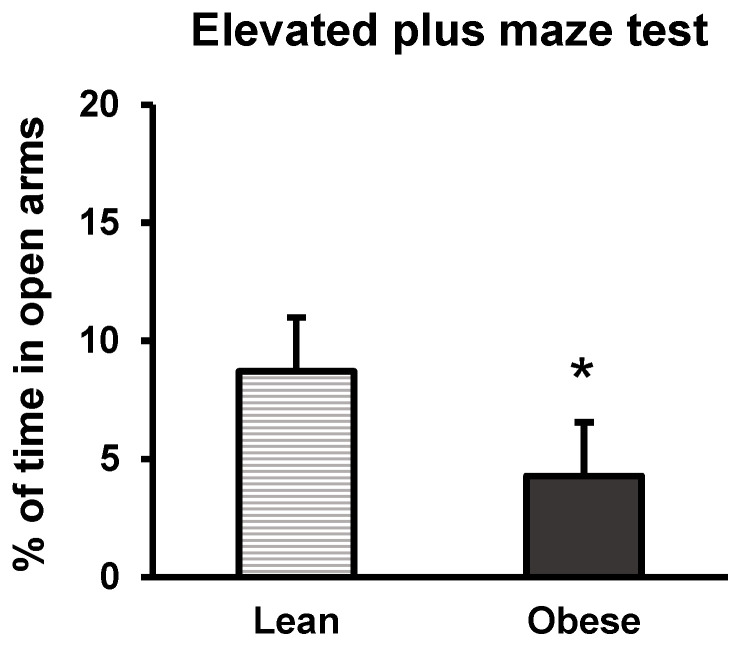
Anxiety-like behaviour in lean and obese mice. Elevated plus maze test. The figure shows the results regarding the open arms of the elevated plus maze test. Percentage of time is represented in relation to the total time (5 min) spent in these arms. Each column represents the mean ± SEM of the values obtained from 16 animals from each group. * *p* < 0.05, compared to the lean group. ES = 0.59 (95% CI for lean: (4.5, 12.9), 95% CI for obese: (0.1, 8.5)).

Anxiety-like behaviour was analysed through the elevated plus maze test. The test is based on the animal’s interaction in each of the arms (closed and open arms). Here, we present the time spent in open arms (with no walls), where the interaction would represent low stress/anxiety levels since the animal feels calm and safe to venture out to explore this arm. This way, results, expressed as the percentage of time spent in the open arms relative to the total time in the test (5 min), showed that obese animals spent less time in this arm compared to lean ones (*p* < 0.05) (Figure 6). Thus, it is suggested that obese animals presented anxiety-like behaviour compared to lean animals.

##### Hole-Board Test

**Figure 7:**
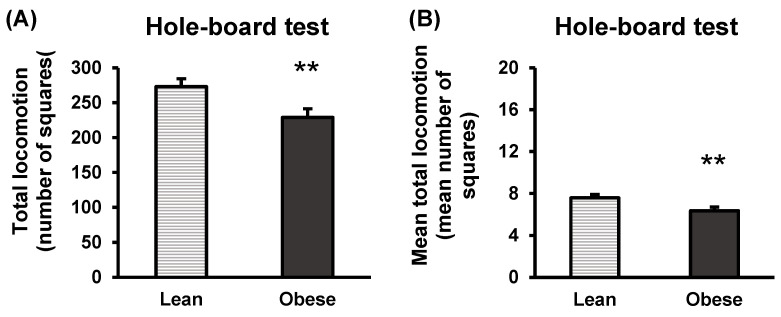
Anxiety-like behaviour in lean and obese mice. Hole-board test. Total locomotion (**A**). Total number of squares crossed during the test duration (5 min) is represented. Mean total locomotion (**B**). The average number of total squares crossed, calculated as the total number of squares crossed divided by the total number of squares (36), is represented. Each column represents the mean ± SEM of the squares crossed by 16 animals of each group. ** *p* < 0.01, compared to the lean group. ES for total locomotion = 1.1 (95% CI for lean: (251.2, 294.8), 95% CI for obese: (205.9, 251.9)); ES for mean total locomotion = 1.1 (95% CI for lean: (7, 8.2), 95% CI for obese: (5.7, 7)).

Figure 7 shows an overview of the results obtained in the hole-board test.

The obese group exhibited statistically significant reduced total locomotion (*p* < 0.01) and mean total locomotion (*p* < 0.01) compared to the lean group.

**Figure 8:**
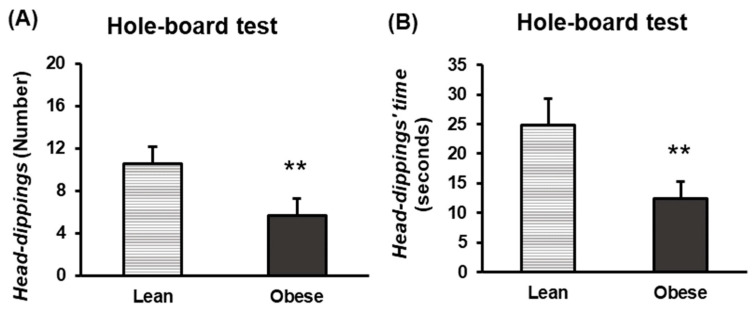
Anxiety-like behaviour in lean and obese mice. Hole-board test. Number of head-dippings (**A**). The number of head-dippings or hole explorations performed during the test duration (5 min) is represented. Time of head-dippings (**B**). The time spent on head-dippings during the test duration (5 min) is represented. Each column represents the mean ± SEM of the values obtained from 16 animals of each group. ** *p* < 0.01, compared to the lean group. ES for number of head-dippings = 0.95 (95% CI for lean: (7.8, 13.4), 95% CI for obese: (2.9, 8.4)); ES for time of head-dippings = 0.98 (95% CI for lean: (16.5, 33.1), 95% CI for obese: (7.1, 17.6)).

Results regarding head-dippings revealed a reduction in both the number (Figure 8A) and duration (Figure 8B) of head-dippings among obese animals (*p* < 0.01), with respect to the lean group, pointing to anxiety-like behaviour in this first group.

Overall, these results indicate that obese animals present anxiety-like behaviour, showcased by lower locomotion as well as a reduction in head-dipping behaviour compared to lean animals.

##### Open Field Test

**Figure 9:**
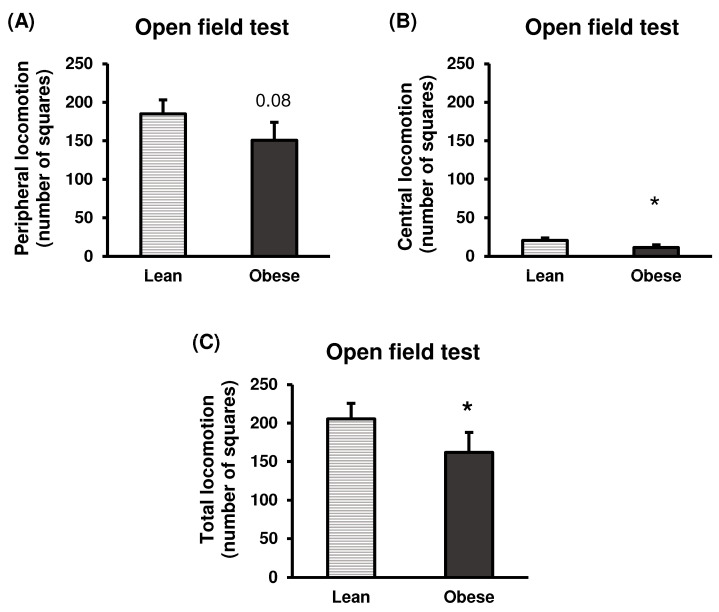
Anxiety-like behaviour in lean and obese mice. Open field test. Peripheral locomotion (**A**). The number of squares crossed in the peripheral zone of the open field during the test duration (5 min) is represented. Central locomotion (**B**). The number of squares crossed in the central zone of the open field during the test duration (5 min) is represented. Total locomotion (**C**). The total number of squares crossed during the test duration (5 min) is represented. Each column represents the mean ± SEM of the number of squares crossed in each zone by 16 animals of each group. * *p* < 0.05, compared to the lean group. ES for peripheral locomotion = 0.5 (95% CI for lean: (152.8, 217.3), 95% CI for obese: (109.7, 191.8)); ES for central locomotion = 0.82 (95% CI for lean: (14.9, 26.4), 95% CI for obese: (5, 17.7)); ES for total locomotion = 0.57 (95% CI for lean: (170.3, 241.1), 95% CI for obese: (116.2, 208)).

Results obtained in the open field test also give information about the animals’ anxiety-like behaviour. Thus, locomotion in the peripheral zone indicates anxiety-like behaviour, while locomotion in the central zone represents a lower level of anxiety, as the animal feels safe enough to go out and explore and browse in an open and spacious area. As shown in Figure 9, obese animals exhibited lower locomotion in the peripheral zone of the open field test (Figure 9A) with respect to lean animals, although without statistically significant differences (*p* = 0.08); while statistically significant lower locomotion in the central area (*p* < 0.05) (Figure 9B), as well as total locomotion (*p* < 0.05) (Figure 9C) were found in this group of obese animals, compared to the lean animals.

Altogether, these results provide important insights into the relationship between obesity and anxiety-like behaviour.

### 3.3. Systemic Biomarkers of the Stress Response

Once a higher level of anxiety in obese animals was verified through behavioural tests, whether these differences also existed in some systematic stress and inflammation markers was assessed.

The table below (Table 3) compares the plasma concentrations of corticosterone, noradrenaline and eHsp72 between lean and obese mice. Obese animals had higher concentrations of corticosterone than lean ones (*p* < 0.05); regarding noradrenaline, obese mice presented reduced concentrations of this hormone (*p* < 0.05). No differences in eHsp72 concentrations between lean and obese animals were detected.

### 3.4. Systemic Inflammatory Cytokines

Table 4 provides information about the plasma concentrations of systemic inflammatory cytokines in lean and obese mice. As shown, an increase in the pro-inflammatory cytokine IL-1β was observed in obese animals compared to lean ones (*p* < 0.05). This cytokine is one of the main inflammatory mediators responsible for amplifying the immune response cascade and contributing to the severity of inflammatory diseases.

However, although a clear trend for increased IL-6 and TNF-α was observed in the obese animals, no significant differences were found in the systemic concentrations of these pro-inflammatory cytokines, and neither in the concentrations of anti-inflammatory cytokine IL-10.

### 3.5. Microbicide Capacity

**Figure 10:**
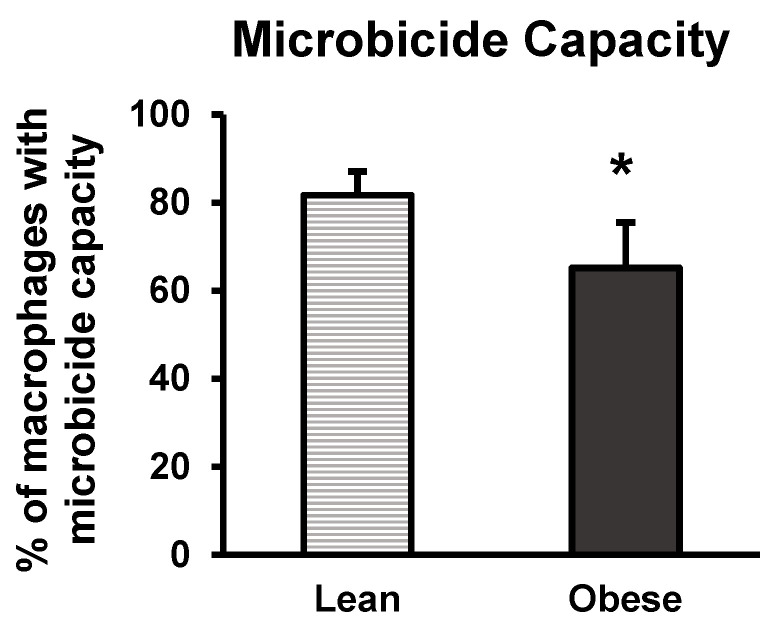
Microbicide percentages of peritoneal macrophages from lean and obese mice. Each column represents the mean ± SEM of the values obtained from 8 animals in each group. Results are expressed in percentages. * *p* < 0.05, compared to the lean group. ES = 0.52 (95% CI for lean: (20.1, 43.4), 95% CI for obese: (15.9, 35)).

Peritoneal macrophages play a crucial role in the immune defence against infections. The microbicide capacity of peritoneal macrophages refers to their ability to destroy or eliminate pathogenic microorganisms, such as bacteria, fungi, or parasites, significantly contributing to the host’s immune response in the presence of infectious agents. Figure 10 shows the results of the oxidative burst assay in lean and obese mice. Obese animals exhibited a reduced microbicide capacity (*p* < 0.05) as represented by the percentage of peritoneal macrophages that produce O_2_^−^ upon the ingestion of bacteria.

### 3.6. WAT Crown-like Structures

**Figure 11:**
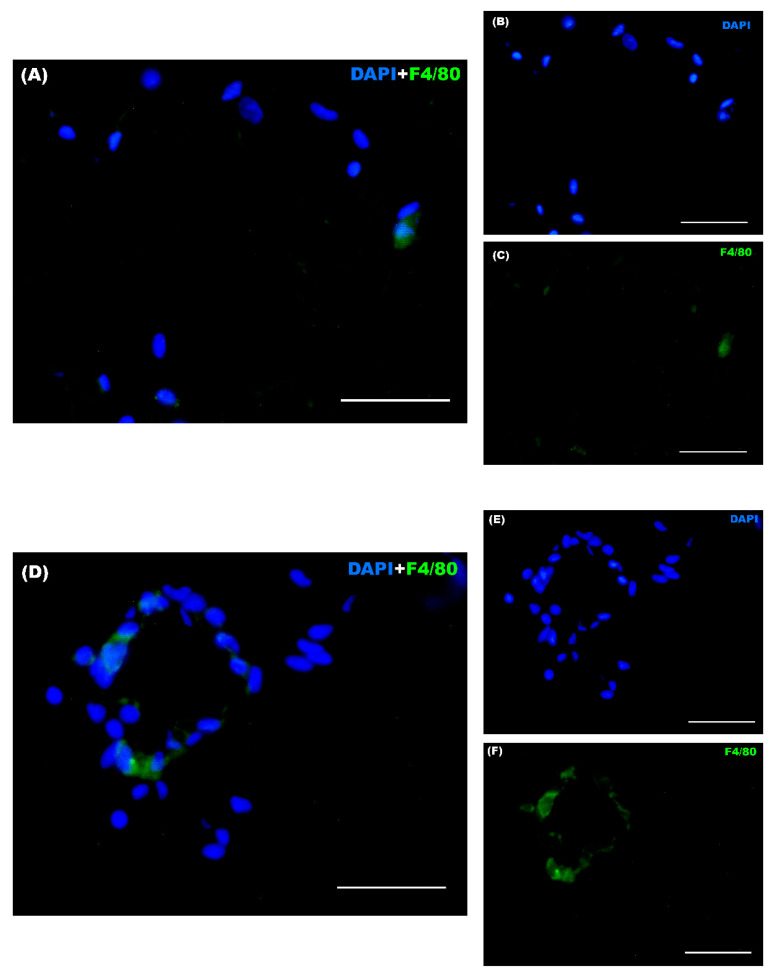
Adipose tissue of lean and obese mice using an immunostaining technique. Lean mouse (**A**). “Crown-like structure” in the adipose tissue of an obese mouse (**D**). Photos were taken under fluorescence microscopy at 40× magnification. Sections were treated with the nuclear stain DAPI (blue) (**B**,**E**) and F4/80 antibody (green) (macrophage marker) (**C**,**F**). Scale bar: 50 μm.

A distinctive trait of macrophages infiltrating adipose tissue is their formation of an aggregate around dead adipocytes that resembles a crown, known as a crown-like structure (CLS), which is observable using immunohistochemical techniques. Table 5 presents the results regarding the formation of these structures within the white adipose tissue, showing a significantly greater number of CLSs in the obese group compared to the lean group (*p* < 0.001) (Figure 11). This result clearly suggests a pro-inflammatory state linked to the formation of these structures, as an indistinguishable hallmark of obesity.

## 4. Discussion

It is well known that obesity represents an immunological and metabolic disorder, with significant implications for physical, psychological, and social well-being [36], associated with comorbidities and metabolic dysfunctions. However, to the best of our knowledge, the present investigation is the first to analyse the interplay between the stress, immune, endocrine, metabolic, and behavioural axis, altogether demonstrating that this relationship is crucial for a better understanding of the pathogenesis and development of this condition, which could enable us to establish optimal strategies for its control and prevention.

As expected, the high-fat diet model proved effective in inducing a state of metabolic obesity, resulting in an increased body weight gain during the 18 weeks of the diet protocol and a higher body weight at the end of the intervention, together with hyperglycaemia and adipocyte hypertrophy, thus indicating a progressive worsening of insulin resistance [37]. Furthermore, cholesterol values were impaired after the diet protocol, and, although differences in triglyceride levels were not found, higher concentrations of this parameter were observed in the group of obese animals. On the other hand, in spite of the impairment of metabolic parameters and the increase in body weight, food intake in obese animals was not higher; in fact, it significantly decreased, probably due to the satiety produced by the hyperlipidic content of the chow administered.

The study of physical condition and behaviour in obese individuals has allowed for a more detailed analysis of this condition, as this disease is not only manifested at the systemic or molecular level but is also reflected in the behaviour of individuals. Some studies have shown that diets enriched in fats and cholesterol can lead to disturbances in the lipid metabolism and disrupted homeostasis, and can also affect brain structures by reducing the volume of the hippocampus. Altogether, this can negatively impact various physiological activities within the brain, including structural development, nerve impulse conduction, neurogenesis, synapse formation, and myelination, as well as behaviour and cognitive functions such as memory, psychomotor efficiency, neural efficiency, attention, and information processing [8,9,10,35]. Thus, in this investigation, a battery of behavioural tests was performed to assess the physical condition, the sensorimotor response, and the anxiety-like behaviour of animals under a high-fat diet experimental protocol.

First, changes in body composition that occur during the development of obesity could worsen physical conditions, and therefore, the strength of the individual [38,39]. One of the changes in body composition of obese individuals is a higher proportion of adipose tissue compared to muscle tissue [40]. This excess fat and its ectopic accumulation, another characteristic of obesity, can interfere with the muscles’ ability to generate force due to the pressure exerted by fat on them [41]. The results presented in this study demonstrate that obesity worsens the physical condition of animals and significantly reduces their strength, indicating that obesity exacerbates this capacity and could be limiting physical activity in obese animals, likely due to the excess adipose tissue and insulin resistance caused by the associated diabetes. Moreover, this loss of strength and, thereby, possible changes in muscle fibre composition due to the loss of lean mass (muscle) and the gain of fat mass, would also be related to the poor motor response found in the obese animals in this study, explaining the worse percentages in the performance and completion of tests assessing both coordination and balance in the obese group of animals. Other studies point out that obesity has been shown to negatively affect the morphology and function of the structures of the vestibular system, responsible for balance and coordination, leading to alterations in these motor systems [42], while it is also thought that structural alterations in several brain regions as a consequence of a state of obesity may be associated with deficits in cognitive and motor domains [43,44]. Thus, obesity affects, directly or indirectly, not only muscle vigour, but also balance and motor coordination, worsening the sensorimotor responses which further complicates the condition of this disease.

We also characterized the global impact of diet and obesity on mice behaviour, particularly assessing the stress response and the anxiety-like behaviour. Regarding the pain response, for example, obese animals presented a trend towards a decrease in latency time, suggesting a higher pain sensitivity compared to lean animals. This reaction could be explained by the alteration of nociceptive perception, as a possible cause of an underlying inflammation, which would produce hyperalgesia in obese animals, so they would react earlier to that pain [45,46].

On the other hand, the rest of the behavioural tests also performed in this study revealed a robust effect of obesity on anxiety-like behaviour, manifested by a lower central, peripheral and total locomotion, together with the presence of behavioural patterns related to anxiety (like head-dippings). Thus, the lower percentage of time in the open arms of the elevated plus maze test, the lower total locomotion in the hole-board test, the lower number and time of head-dippings and the lower central and total locomotion in the open field test performed by obese mice, altogether, provide definitive evidence of an anxiety-like behaviour related to the obese state.

All these tests are based on thigmotaxis behaviour, the tendency of rodents to explore and move along the edges of a space, rather than directly across it. When a rodent is in a new or unfamiliar environment, it tends to feel more secure and comfortable when in contact with surfaces, such as walls or other objects. Thigmotaxis is a natural, adaptive behaviour that can help rodents feel protected and orient themselves in their environment [47]. By maintaining constant contact with surfaces, they can obtain tactile and spatial information, allowing them to avoid obstacles, find shelter, and locate resources such as food or hiding places. It is in this type of situation that we refer that the animal’s state of anxiety as higher, as it feels potentially threatened and therefore tends to seek shelter. On the contrary, when the animal’s state is calm, it may go to the surface and more open areas in order to browse, showing less anxious and fearful behaviour. The lack of this last behaviour was noted in our battery of tests, when the animals reduced their time in the open arms or locomotion in central zones and in total, depicting a state of anxiety in those animals. In the case of the hole-board test, the reduction in the number of crossed squares is associated with freezing behaviour, one of the behavioural patterns performed by an anxious animal [48], since it remains stationary in a particular area of the board, thereby reducing its locomotion. Finally, other patterns that reflect an anxiety state are the frequency and time of head-dippings (analysed in the hole-board test too), which were reduced in obese mice, and are considered inversely proportional to the animal’s anxiety state [32]. In this model of obesity induced by a hyperlipidic diet, it is clear that obesity produces stress and anxiety-like behaviour, in addition to poor motor coordination and balance response.

Additionally, systemic stress biomarkers were also assessed. Corticosterone is the main glucocorticoid released by rodents, involved in the regulation of metabolism, immune, and stress response [49,50]. The fact that obese animals show a high concentration of systemic corticosterone could be reflecting the presence of chronic stress, since when a stressor is sustained over time, glucocorticoid levels remain sufficiently elevated to recruit the network of responses to that stress [51]. These results, together with the ones demonstrating the anxiety-like behaviour in obese mice, would be supporting this behaviour quantitatively through a stress biomarker, in this case corticosterone, reinforcing the hypothesis that obesity can lead to a state of chronic stress/anxiety in individuals suffering from this disease.

Regarding noradrenaline, a decrease in the systemic concentrations of this neurotransmitter was observed in obese mice. This result is in agreement with those obtained in humans and rodents with obesity [52,53,54]. It is known that noradrenaline plays a crucial role in the regulation of body weight and energy metabolism. These hormones bind to cells that carry adrenergic receptors, thus stimulating the breakdown of fat, and releasing energy [55]. However, obesity provokes alterations in noradrenergic functions [56,57], such as changes in the sensitivity and expression of adrenergic receptors [58] which could induce resistance to catecholamines by adipocytes [53]. However, this noradrenergic dysregulation manifests itself differently in different types of obesity [56,57], sometimes by increasing circulating levels of noradrenaline [58]. For example, in a Zucker rat model of genetic obesity, we observed an overactivity of the sympathetic nervous system that generated an increase in systemic concentrations of NA, also related to the inflammatory state of the animals [58]. Therefore, the decrease in systemic noradrenaline concentrations found in the obese animals in the present study could be due to a specific noradrenergic dysregulation associated with metabolic and inflammatory alterations in high-fat diet-induced obesity.

Thus, when we evaluated the inflammatory response, in parallel to the systemic stress response, elevated circulating concentrations of IL-1β were found in the present high-fat diet-induced obesity model. IL-1β, which is mainly produced by macrophages, is one of the key inflammatory cytokines responsible for amplifying the immune response cascade and contributing to the severity of inflammatory diseases [59,60,61]. Additionally, in the context of immunoneuroendocrine interaction, IL-1β activates the hypothalamic-pituitary-adrenal (HPA) axis for the release of glucocorticoids, according to the results herein observed. This physiological increase of corticosterone induced by the pro-inflammatory cytokine IL-1β has physiological relevance for preventing organisms from an excessive inflammatory response [62], and it seems to be altered in the high-fat diet-induced obese animals. Taking into account that NA inhibits the macrophage release of IL-1β [63,64], the decrease in the NA concentration is also consistent with the increase in the IL-1β systemic concentration.

The adrenergic regulation of the immune system, and particularly of the macrophage-mediated innate/inflammatory immune response, depends on the activity of the sympathetic nervous system (SNS) and the hypothalamic-pituitary-adrenal (HPA) axis, and obesity is a condition that presents immunological, sympathetic activity, and HPA axis changes [65]. The dysregulated inmunoneuroendocrine interaction mediated by cytokine-induced inflammatory response and the SNS and HHA axis seems also dependent on the type of obesity and their metabolic and inflammatory dysfunction. Previous results from our research group demonstrated, in the genetic model of metabolic syndrome (Zucker rat), that increased NA has an inhibitory role in the release of IL-1β and stimulates the release of IL-6 by peritoneal macrophages [66]. Thus, the decrease in NA concentration explains the increase in the IL-1β and the absence of significant modifications in the concentration of IL-6 in the present model of obesity. In addition, while an alteration of the IL-6/NA feedback and impairment in the NA-mediated regulation of cytokines released by peritoneal macrophages are involved in genetic-induced obesity [58,66], the dysregulation between the IL-1β-mediated inflammatory response and corticosterone-mediated stress response is involved in the high-fat diet-induced obesity.

In addition to generating stress and anxiety-like behaviour, obesity causes a worsened innate immune response, the first defence mechanism against attack by pathogens based on the action of phagocytic cells (mainly neutrophils and macrophages) and products released by these cells, including inflammatory cytokines. It has been shown that obese animals present a worsened innate immune response in circulating monocytes [67] and peritoneal macrophages [68] compared to lean ones. For this study, peritoneal macrophages were used for assessing the microbicide capacity of obese animals, since those cells are the most representative macrophages among all macrophage populations in the body, making them ideal for assessing the activation status of this cell type. Obese animals in this study were found to have a reduced microbicide capacity, reflecting a weakened innate immune response that can underlie the greater susceptibility to infections reported in obese individuals [69]. Since NA can stimulate [70] and glucocorticoids inhibit [71,72] the microbicide capacity of phagocytes, differences in the concentrations of corticosterone and NA between obese and lean animals in the present study are compatible with the results obtained regarding the microbicide capacity of peritoneal macrophages.

Moreover, adipose tissue is crucial in regulating systemic energy homeostasis and assumes a pivotal function in the regulation and maintenance of a state of obesity, in which inflammation and macrophages also play an important role. During obesity, WAT can undergo severe dysfunction, impeding its proper expansion to store excess energy [73]. This is because during overnutrition, mature adipocytes accumulate more fat and, as a result, there is an increase in the size of adipocytes (hypertrophy), and/or the number of adipocytes (hyperplasia). When assessing this tissue, it was clear that the adipocytes from the obese group present hypertrophy, exhibiting a much larger size compared to the lean group. These results are important, since it is known that in healthy conditions, WAT, in addition to being the main fat storage depot, has endocrine functions by secreting adipokines and cytokines systemically [74]. Thus, obesity causes a phenotypic change in this tissue, characterized by the appearance of inflamed and dysfunctional adipocytes that locally and systemically secrete pro-inflammatory cytokines that disrupt the normal function of the adipose tissue. Macrophage infiltration is responsible for the development and maintenance of obesity-induced inflammation, and for many pro-inflammatory molecules secreted by this tissue [36,75]. A characteristic feature of these macrophages is that they form a crown-like aggregate around dead adipocytes, known as a crown-like structure (CLS). These crown-shaped structures form around necrotic adipocytes because once the cell is dead it would have to be removed. The results obtained in the present work are in line with those of previous studies, as well as those of our research group [20], showing a significant increase in the number of CLSs in the group of obese mice, compared to the lean animals, confirming the presence of these structures more frequently in the obese group, and, therefore, a greater number of necrotic adipocytes, due to obesity and its underlying inflammation.

Considering the severity and frequency of this disease, these results can contribute to a better understanding of the interplay between the state of obesity and the broad immunoneuroendocrine, metabolic, behavioural, and inflammatory axis, measured for the first time together in the same model of animal obesity, with the aim of gaining deeper insights into the pathophysiological implications of this disease and its comorbidities in order to address possible preventive measures and new effective intervention strategies for this syndrome.

## 5. Conclusions

High-fat diet-induced obesity with an impaired metabolic status (glucose and lipid metabolism) provokes a worsened physical condition, poor motor coordination and balance, the presence of anxiety-like behaviour and stress, and a weakened innate immune response. Inmunoneuroendocrine (the interaction between the inflammatory response mediated by IL-1β and the stress response mediated by corticosterone) and noradrenergic dysregulation, and infiltrated inflammatory macrophages are features that underlie these effects. It is essential to clearly understand these interactions when developing potential strategies and approaches for the control and management of obesity. Thus, in the clinical practice involving dietary strategies for obesity (with or without adequate and non-detrimental exercise programs), it seems imperative to avoid or reduce anxiety and stress in order to achieve effective metabolic and immunophysiological benefits.

## Figures and Tables

**Table 1:** Body weights, food intake, glycaemic and lipid profiles in lean and obese mice.

	Lean	Obese	*p*-value	ES
Body weight (g)	23.8 ± 1.8 (22.2, 25.3)	39.1 ± 8.5 (30.2, 48) ***	*p* = 0.0001 ***	2.7
Adipocyte size (μm)	58.8 ± 2.4 (51.1, 66.5)	105 ± 2.4 (79.7, 131.2) ***	*p* = 0.0007 ***	3.9
Food intake (g/day)	3.5 ± 0.08 (3.4, 3.5)	3 ± 0.3 (2.8, 3.1) ***	*p* = 3.2392 × 10^−9^	2.6
Glucose (mg/dL)	173 ± 51 (130.4, 215.6)	247.3 ± 96.2 (146.4, 348.39) *	*p* = 0.02 *	1
Cholesterol (mg/dL)				
- Total Cholesterol	<100	198 ± 88.6 (88, 308) *	*p* = 0.03 *	1.4
- HDL Cholesterol	50.5 ± 7.1 (32.3, 68.7)	>80 *	*p* = 0.02 *	1.9
- LDLc Cholesterol	32 ± 7.3 (14.2, 49.8)	90 ± 32.9 (5.3, 174.5) *	*p* = 0.04 *	1
Triglycerides (mg/dL)	<50	88.6 ± 14 (49.7, 127.5)	*p* = 0.16	0.7

Each value represents the mean ± SEM (Confidence Interval 95%) of 8 animals in each group for metabolic parameters, and in 4 animals in each group for adipocyte size in 10 randomly selected fields. ES: effect size. HDL—high-density lipoproteins. LDL—low-density lipoproteins. LCLc, LDL calculated using the Friedewald equation (LDL = Total cholesterol − (Triglycerides/5) − HDL). * *p* < 0.05, *** *p* < 0.001 compared to the lean group.

**Table 2:** Pain response to nociceptive stimuli in lean and obese mice.

Hot Plate Test	Lean	Obese	*p*-Value	ES
Pain threshold (°C)	46.4 ± 0.7 (45, 47.7)	46.8 ± 0.3 (46.3, 47.2)	*p* = 0.27	0.22
Pain latency (s)	5.1 ± 0.4 (4.3, 5.8)	4.2 ± 0.6 (3.2, 5.2)	*p* = 0.07	0.52

Each value represents the mean ± SEM (Confidence Interval 95%) of the values determined in 16 animals of each group. Results are expressed in degrees Celsius or seconds, respectively. ES: effect size.

**Table 3:** Plasma concentrations of systemic biomarkers of the stress response of lean and obese animals.

	Lean	Obese	*p*-Value	ES
Corticosterone (ng/mL)	1390 ± 282 (1040.1, 1740.1)	1598.2 ± 42.3 (1545.6, 1650.7) *	*p* = 0.04 *	1.03
Noradrenaline (pg/mL)	11.69 ± 6 (2.2, 21.2)	7.85 ± 2 (5.5, 10.2) *	*p* = 0.05	0.92
eHsp72 (ng/mL)	1.68 ± 1.4 (−0.1, 3.5)	1.49 ± 0.2 (1.3, 1.7)	*p* = 0.4	0.18

Each value represents the mean ± SEM (Confidence Interval 95%) of the values obtained from 5 animals in each group. Results are expressed in pg/mL or ng/mL. * *p* ≤ 0.05 compared to the lean group. ES: effect size.

**Table 4:** Plasma concentrations of systemic inflammatory cytokines of lean and obese mice.

	Lean	Obese	*p*-Value	ES
IL-1β	10.4 ± 2 (4.8, 16.1)	13.5 ± 0 (13.5, 13.6) *	*p* = 0.04 *	0.97
IL-10	22.5 ± 5.6 (7, 38)	25.3 ± 9.7 (−1.7, 52.3)	*p* = 0.39	0.16
IL-6	15 ± 2.2 (8.7, 21.2)	18.1 ± 3.1 (9.4, 26.8)	*p* = 0.23	0.5
TNF-α	14.3 ± 4.1 (2.8, 25.8)	17.8 ± 4.4 (3.6, 27.9)	*p* = 0.41	0.2

Each value represents the mean ± SEM (Confidence Interval 95%) of the values obtained from 5 animals in each group. Results are expressed in pg/mL. * *p* < 0.05 compared to the lean group. ES: effect size.

**Table 5:** Number of crown-like structures found in the adipose tissue of lean and obese mice.

	Lean	Obese	*p*-Value	ES
CLS	0.2 ± 0.2 (−0.4, 0.8)	3.8 ± 0.6 (2.2, 5.4) ***	*p* = 0.0002	3.7

Each value represents the mean ± SEM (Confidence Interval 95%) of the values found in the adipose tissue of 5 animals in each group, through the use of fluorescence microscopy in 10 randomly selected fields at 40× magnification. *** *p* < 0.001 compared to the lean group. ES: effect size.

## Data Availability

The original contributions presented in the study are included in the article, further inquiries can be directed to the corresponding author/s.

## References

[1] Speakman J.R. (2019). Use of high-fat diets to study rodent obesity as a model of human obesity. Int. J. Obes..

[2] Amiri S., Behnezhad S. (2019). Obesity and anxiety symptoms: A systematic review and meta-analysis. Neuropsychiatrie.

[3] Coccaro E.F., Lee R., Coussons-Read M. (2014). Elevated plasma inflammatory markers in individuals with intermittent explosive disorder and correlation with aggression in humans. JAMA Psychiatry.

[4] Hilakivi-Clarke L., Cho E., Onojafe I. (1996). High-fat diet induces aggressive behavior in male mice and rats. Life Sci..

[5] Veniaminova E., Cespuglio R., Markova N., Mortimer N., Cheung C.W., Steinbusch H.W., Lesch K.-P., Strekalova T. (2016). Behavioral Features of Mice Fed with a Cholesterol-Enriched Diet: Deficient Novelty Exploration and Unaltered Aggressive Behavior. Transl. Neurosci. Clin..

[6] Sharma S., Fulton S. (2013). Diet-induced obesity promotes depressive-like behaviour that is associated with neural adaptations in brain reward circuitry. Int. J. Obes..

[7] Takase K., Tsuneoka Y., Oda S., Kuroda M., Funato H. (2016). High-fat diet feeding alters olfactory-, social-, and reward-related behaviors of mice independent of obesity. Obesity.

[8] Oliveira T.G., Chan R.B., Bravo F.V., Miranda A., Silva R.R., Zhou B., Marques F., Pinto V., Cerqueira J.J., Di Paolo G. (2015). The impact of chronic stress on the rat brain lipidome. Mol. Psychiatry.

[9] Peng K.Y., Pérez-González R., Alldred M.J., Goulbourne C.N., Morales-Corraliza J., Saito M., Saito M., Ginsberg S.D., Mathews P.M., Levy E. (2019). Apolipoprotein E4 genotype compromises brain exosome production. Brain.

[10] Xu J., Gao H., Zhang L., Rong S., Yang W., Ma C., Chen M., Huang Q., Deng Q., Huang F. (2019). Melatonin alleviates cognition impairment by antagonizing brain insulin resistance in aged rats fed a high-fat diet. J. Pineal Res..

[11] Das U.N. (2001). Is obesity an inflammatory condition?. Nutrition.

[12] Luppino F.S., De Wit L.M., Bouvy P.F., Stijnen T., Cuijpers P., Penninx B.W.J.H., Zitman F.G. (2010). Overweight, Obesity, and Depression: A Systematic Review and Meta-analysis of Longitudinal Studies. Arch. Gen. Psychiatry.

[13] Harrison N.A., Cercignani M., Voon V., Critchley H.D. (2014). Effects of Inflammation on Hippocampus and Substantia Nigra Responses to Novelty in Healthy Human Participants. Neuropsychopharmacology.

[14] Haba R., Shintani N., Onaka Y., Wang H., Takenaga R., Hayata A., Baba A., Hashimoto H. (2012). Lipopolysaccharide affects exploratory behaviors toward novel objects by impairing cognition and/or motivation in mice: Possible role of activation of the central amygdala. Behav. Brain Res..

[15] Augustsson H., Dahlborn K., Meyerson B.J. (2005). Exploration and risk assessment in female wild house mice (Mus musculus musculus) and two laboratory strains. Physiol. Behav..

[16] Zhu Y.C., Wu S.K., Cairney J. (2011). Obesity and motor coordination ability in Taiwanese children with and without developmental coordination disorder. Res. Dev. Disabil..

[17] Cheng J., East P., Blanco E., Kang Sim E., Castillo M., Lozoff B., Gahagan S. (2016). Obesity leads to declines in motor skills acrosschildhood. Child. Care. Health Dev..

[18] Seebacher F., Tallis J., Mcshea K., James R.S. (2017). Obesity-induced decreases in muscle performance are not reversed by weight loss. Int. J. Obes..

[19] Morrison C.D., Pistell P.J., Ingram D.K., Johnson W.D., Liu Y., Fernandez-Kim S.O., White C.L., Purpera M.N., Uranga R.M., Bruce-Keller A.J. (2010). High fat diet increases hippocampal oxidative stress and cognitive impairment in aged mice: Implications for decreased Nrf2 signaling. J. Neurochem..

[20] Gálvez I., Hinchado M.D., Martín-Cordero L., Morán-Plata F.J., Graham G., Francisco-Morcillo J., Ortega E. (2023). The anti-inflammatory and bioregulatory effects of habitual exercise in high-fat diet-induced obesity involve crown-like structures and MCP-1 in white adipose tissue. Exerc. Immunol. Rev..

[21] Martín-Cordero L., Gálvez I., Hinchado M.D., Ortega E. (2020). Influence of Obesity and Exercise on β2-Adrenergic-Mediated Anti-Inflammatory Effects in Peritoneal Murine Macrophages. Biomedicines.

[22] Saltiel A.R., Olefsky J.M. (2017). Inflammatory mechanisms linking obesity and metabolic disease. J. Clin. Investig..

[23] Gálvez I., Martín-Cordero L., Hinchado M.D., Álvarez-Barrientos A., Ortega E. (2019). Anti-inflammatory effect of β2 adrenergic stimulation on circulating monocytes with a pro-inflammatory state in high-fat diet-induced obesity. Brain. Behav. Immun..

[24] Chawla A., Nguyen K.D., Goh Y.P.S. (2011). Macrophage-mediated inflammation in metabolic disease. Nat. Rev. Immunol..

[25] Haase J., Weyer U., Immig K., Klöting N., Blüher M., Eilers J., Bechmann I., Gericke M. (2014). Local proliferation of macrophages in adipose tissue during obesity-induced inflammation. Diabetologia.

[26] Kawai T., Autieri M.V., Scalia R. (2021). Adipose tissue inflammation and metabolic dysfunction in obesity. Am. J. Physiol. Cell Physiol..

[27] Cabe P.A., Tilson H.A., Mitchell C.L., Dennis R. (1978). A Simple Recording Grip Strength Device 1. Pharmacol. Biochem. Behav..

[28] Baeza I., De Castro N.M., Giménez-Llort L., De la Fuente M. (2010). Ovariectomy, a model of menopause in rodents, causes a premature aging of the nervous and immune systems. J. Neuroimmunol..

[29] Himanshu, Dharmila, Sarkar D., Nutan (2020). A review of behavioral tests to evaluate different types of anxiety and anti-anxiety effects. Clin. Psychopharmacol. Neurosci..

[30] Walf A.A., Frye C.A. (2007). The use of the elevated plus maze as an assay of anxiety-related behavior in rodents. Nat. Protoc..

[31] Viveros M.P., Fernández B., Guayerbas N., De la Fuente M. (2001). Behavioral characterization of a mouse model of premature immunosenescence. J. Neuroimmunol..

[32] File S.E., Wardill A.G. (1975). Validity of head-dipping as a measure of exploration in a modified hole-board. Psychopharmacologia.

[33] de Cabo de la Vega C., Pujol A., Viveros M.P. (1995). Neonatally administered naltrexone affects several behavioral responses in adult rats of both genders. Pharmacol. Biochem. Behav..

[34] Albonetti M.E., Farabollini F. (1992). Behavioural responses to single and repeated restraint in male and female rats. Behav. Process..

[35] Abbott K.N., Arnott C.K., Westbrook R.F., Tran D.M.D. (2019). The effect of high fat, high sugar, and combined high fat-high sugar diets on spatial learning and memory in rodents: A meta-analysis. Neurosci. Biobehav. Rev..

[36] Basdevant A. (2006). L’obésité: Origines et conséquences d’une épidémie. Comptes Rendus Biol..

[37] Jellinger P.S. (2007). Metabolic consequences of hyperglycemia and insulin resistance. Clin. Cornerstone.

[38] Liou T.H., Pi-Sunyer F.X., Laferrère B. (2005). Physical disability and obesity. Nutr. Rev..

[39] Tomlinson D.J., Erskine R.M., Morse C.I., Winwood K., Onambélé-Pearson G. (2016). The impact of obesity on skeletal muscle strength and structure through adolescence to old age. Biogerontology.

[40] Visser M., Kritchevsky S.B., Goodpaster B.H., Newman A.B., Nevitt M., Elizabeth S., Harris T.B. (2002). Leg Muscle Mass and Composition in Relation to Lower Extremity Performance in Men and Women Aged 70 to 79: The Health, Aging and Body Composition Study. J. Am. Geriatr. Soc..

[41] Roy B., Curtis M.E., Fears L.S., Nahashon S.N., Fentress H.M. (2016). Molecular mechanisms of obesity-induced osteoporosis and muscle atrophy. Front. Physiol..

[42] Kawao N., Takafuji Y., Ishida M., Okumoto K., Morita H., Muratani M., Kaji H. (2020). Roles of the vestibular system in obesity and impaired glucose metabolism in high-fat diet-fed mice. PLoS ONE.

[43] Bolzenius J.D., Laidlaw D.H., Cabeen R.P., Conturo T.E., McMichael A.R., Lane E.M., Heaps J.M., Salminen L.E., Baker L.M., Scott S.E. (2015). Brain structure and cognitive correlates of body mass index in healthy older adults. Behav. Brain Res..

[44] Walther K., Birdsill A.C., Glisky E.L., Ryan L. (2010). Structural brain differences and cognitive functioning related to body mass index in older females. Hum. Brain Mapp..

[45] Wang C., Chan J.S.Y., Ren L., Yan J.H. (2016). Obesity Reduces Cognitive and Motor Functions across the Lifespan. Neural Plast..

[46] Matsuda M., Huh Y., Ji R.R. (2019). Roles of inflammation, neurogenic inflammation, and neuroinflammation in pain. J. Anesth..

[47] Simon P., Dupuis R., Costentin J. (1994). Thigmotaxis as an index of anxiety in mice. Influence of dopaminergic transmissions. Behav. Brain Res..

[48] Weiss S.M., Lightowler S., Stanhope K.J., Kennett G.A., Dourish C.T. (2000). Measurement of anxiety in transgenic mice. Rev. Neurosci..

[49] Cain D.W., Cidlowski J.A. (2017). Immune regulation by glucocorticoids. Nat. Rev. Immunol..

[50] Nicolaides N.C., Kyratzi E., Lamprokostopoulou A., Chrousos G.P., Charmandari E. (2014). Stress, the Stress System and the Role of Glucocorticoids. Neuroimmunomodulation.

[51] Dallman M.F., Pecoraro N.C., La Fleur S.E., Warne J.P., Ginsberg B., Akana S.F., Laugero K.C., Houshyar H., Strack A.M., Bhatnagar S. (2006). Glucocorticoids, chronic stress, and obesity. Prog. Brain Res..

[52] Astrupl A.V., Christensen N.J., Leif B. (1991). Reduced plasma noradrenaline concentrations in simple- obese and diabetic obese patients. Clin. Sci..

[53] Guo T., Marmol P., Moliner A., Björnholm M., Zhang C., Shokat K.M., Ibanez C.F. (2014). Adipocyte ALK7 links nutrient overload to catecholamine resistance in obesity. eLife.

[54] Qi Z., Ding S. (2015). Obesity-associated sympathetic overactivity in children and adolescents: The role of catecholamine resistance in lipid metabolism. J. Pediatr. Endocrinol. Metab..

[55] Thorp A.A., Schlaich M.P. (2015). Relevance of Sympathetic Nervous System Activation in Obesity and Metabolic Syndrome. J. Diabetes Res..

[56] Straznicky N.E., Lambert G.W., Lambert E.A. (2010). Noradrenergic dysfunction in obesity: An overview of the effects of weight loss. Curr. Opin. Lipidol..

[57] Straznicky N.E., Lambert E.A., Nestel P.J., McGrane M.T., Dawood T., Schlaich M.P., Masuo K., Eikelis N., De Courten B., Mariani J.A. (2010). Sympathetic neural adaptation to hypocaloric diet with or without exercise training in obese metabolic syndrome subjects. Diabetes.

[58] Martín-Cordero L., García J.J., Hinchado M.D., Ortega E. (2011). The interleukin-6 and noradrenaline mediated inflammation-stress feedback mechanism is dysregulated in metabolic syndrome: Effect of exercise. Cardiovasc. Diabetol..

[59] Abbate A., Canada J.M., Van Tassell B.W., Wise C.M., Dinarello C.A. (2014). Interleukin-1 blockade in rheumatoid arthritis and heart failure: A missed opportunity?. Int. J. Cardiol..

[60] Dinarello C.A., van der Meer J.W.M. (2013). Treating inflammation by blocking interleukin-1 in humans. Semin. Immunol..

[61] Dinarello C.A. (2014). An expanding role for interleukin-1 blockade from gout to cancer. Mol. Med..

[62] Besedovsky H.O., del Rey A. (2007). Processing of Cytokine Signals at CNS Levels. Relevance for Immune-HPA Axis Interactions. NeuroImmune Biol..

[63] Elenkov I.J., Chrousos G.P. (2002). Stress hormones, proinflammatory and antiinflammatory cytokines, and autoimmunity. Ann. N. Y. Acad. Sci..

[64] Elenkov I.J., Iezzoni D.G., Daly A., Harris A.G., Chrousos G.P. (2005). Cytokine dysregulation, inflammation and well-being. Neuroimmunomodulation.

[65] Ortega E., Gálvez I., Martín-Cordero L. (2019). Adrenergic Regulation of Macrophage-Mediated Innate/Inflammatory Responses in Obesity and Exercise in this Condition: Role of β2 Adrenergic Receptors. Endocr. Metab. Immune Disord. Drug Targets.

[66] Martín-Cordero L., García J.J., Ortega E. (2013). Noradrenaline-mediated inhibition of inflammatory cytokines is altered in macrophages from obese Zucker rats: Effect of habitual exercise. Endocr. Metab. Immune Disord. Drug Targets.

[67] Gálvez I., Martín-Cordero L., Hinchado M.D., Ortega E. (2020). β2 Adrenergic Regulation of the Phagocytic and Microbicide Capacity of Circulating Monocytes: Influence of Obesity and Exercise. Nutrients.

[68] Martín-Cordero L., Reis F., Garcia J.J., Teixeira F., Ortega E. (2013). Effect of exercise without diet on functional capacity of peritoneal macrophages and TNF-a levels in blood and in adipose tissue in the obese Zucker rat model of the metabolic syndrome. Proc. Nutr. Soc..

[69] Ortega E., Martín-Cordero L., Garcia-Roves P.M., Chicco A.J., González-Franquesa A., Marado D., Palavra F., Reis F., Marado D., Sena A. (2015). Diabetes Mellitus and Metabolic Syndrome. Biomarkers of Cardiometabolic Risk, Inflammation and Disease.

[70] Ortega E., Giraldo E., Hinchado M.D., Martín L., García J.J., la Fuente M.D. (2007). Neuroimmunomodulation during Exercise: Role of Catecholamines as ‘Stress Mediator’ and/or ‘Danger Signal’ for the Innate Immune Response. Neuroimmunomodulation.

[71] Wang J., Wang R., Wang H., Yang X., Yang J., Xiong W., Wen Q., Ma L. (2017). Glucocorticoids Suppress Antimicrobial Autophagy and Nitric Oxide Production and Facilitate Mycobacterial Survival in Macrophages. Sci. Rep..

[72] Schaffner A. (1985). Therapeutic concentrations of glucocorticoids suppress the antimicrobial activity of human macrophages without impairing their responsiveness to gamma interferon. J. Clin. Investig..

[73] Longo M., Zatterale F., Naderi J., Parrillo L., Formisano P., Raciti G.A., Beguinot F., Miele C. (2019). Adipose Tissue Dysfunction as Determinant of Obesity-Associated Metabolic Complications. Int. J. Mol. Sci..

[74] Musi N., Guardado-Mendoza R. (2014). Adipose Tissue as an Endocrine Organ. Cellular Endocrinology in Health and Disease.

[75] McArdle M.A., Finucane O.M., Connaughton R.M., McMorrow A.M., Roche H.M. (2013). Mechanisms of obesity-induced inflammation and insulin resistance: Insights into the emerging role of nutritional strategies. Front. Endocrinol..

